# On the System of High Order Rational Difference Equations

**DOI:** 10.1155/2014/760502

**Published:** 2014-10-29

**Authors:** Qianhong Zhang, Wenzhuan Zhang, Yuanfu Shao, Jingzhong Liu

**Affiliations:** ^1^Guizhou Key Laboratory of Economics System Simulation, Guizhou University of Finance and Economics, Guiyang, Guizhou 550004, China; ^2^School of Science, Guilin University of Technology, Guilin, Guangxi 541000, China; ^3^Department of Mathematics and Physics, Hunan Institute of Technology, Hengyang, Hunan 421002, China

## Abstract

This paper is concerned with the boundedness, persistence, and global asymptotic behavior of positive solution for a system of two rational difference equations *x*_*n*+1_ = *A* + (*x*_*n*_/∑_*i*=1_^*k*^ *y*_*n*−*i*_), *y*_*n*+1_ = *B* + (*y*_*n*_/∑_*i*=1_^*k*^ *x*_*n*−*i*_),  *n* = 0,1,…, *k* ∈ {1,2,…}, where *A*, *B* ∈ (0, ∞), *x*_−*i*_ ∈ (0, ∞), and *y*_−*i*_ ∈ (0, ∞), *i* = 0,1, 2,…, *k*.

## 1. Introduction

In this paper, we study the global behavior of solutions of the following system:

(1) xn+1=A+xn∑i=1kyn−i,  yn+1=B+yn∑i=1kxn−i,n=0,1,…,

where *A*, *B* are positive constants and initial conditions *x*_−*i*_, *y*_−*i*_ ∈ (0, *∞*),  *i* = 0,1, 2,…, *k*.

A pair of sequences of positive real numbers {(*x*_*n*_, *y*_*n*_)} that satisfies (1) is a positive solution of (1). If a positive solution of (1) is a pair of positive constants (*x*, *y*), then the solution is the equilibrium solution.

A positive solution {(*x*_*n*_, *y*_*n*_)} of (1) is bounded and persists, if there exist positive constants *M*, *N* such that

(2) $$\begin{array}{c} M\leq x_{n},\;y_{n}\leq N,\;n=-2,-1,\ldots . \end{array}$$

In 1998, DeVault et al. [1] proved that every positive solution of the difference equation

(3) $$\begin{array}{c} x_{n+1}=A+\frac{x_{n}}{x_{n-1}},\;n=0,1,\ldots , \end{array}$$

where *A* ∈ (0, *∞*), oscillates about the positive equilibrium *c* = 1 + *A* of (3). Moreover, every positive solution of (3) is bounded away from zero and infinity. Also the positive equilibrium of (3) is globally asymptotically stable.

In 2003, Abu-Saris and DeVault [2] studied the following recursive difference equation:

(4) $$\begin{array}{c} x_{n+1}=A+\frac{x_{n}}{x_{n-k}},\;n=0,1,\ldots , \end{array}$$

where *A* ∈ (1, +*∞*),  *x*_−*k*_, *x*_−*k*+1_,…, *x*_0_ are positive real numbers.

Papaschinopoulos and Schinas [3] investigated the global behavior for a system of the following two nonlinear difference equations:

(5) xn+1=A+ynxn−p,  yn+1=A+xnyn−q,n=0,1,…,

where *A* is a positive real number, *p*, *q* are positive integers, and *x*_−*p*_,…, *x*_0_,  *y*_−*q*_,…, *y*_0_ are positive real numbers.

In 2012, Zhang et al. [4] investigated the global behavior for a system of the following third-order nonlinear difference equations:

(6) $$\begin{array}{c} x_{n+1}=A+\frac{x_{n}}{y_{n-1}+y_{n-2}},\;\;y_{n+1}=B+\frac{y_{n}}{x_{n-1}+x_{n-2}}, \end{array}$$

where *A*, *B* ∈ (0, *∞*), and the initial values *x*_−*i*_, *y*_−*i*_ ∈ (0, *∞*),  *i* = 0,1. For other related results, the reader can refer to [5–18].

Motivated by the discussion above, we study the global asymptotic behavior of solutions for system (1). More precisely, we prove the following: if *A* > 1/*k*, *B* > 1/*k* then every positive solution {(*x*_*n*_, *y*_*n*_)} of (1) is persistent and bounded. Moreover, we prove that every positive solution {(*x*_*n*_, *y*_*n*_)} of (1) converges the unique positive equilibrium (*x*, *y*) as *n* → *∞*.

## 2. Main Results

In the following lemma, we show boundedness and persistence of the positive solutions of (1).

**Lemma 1 .**

Consider (1). Suppose that

(7) $$\begin{array}{c} A>\frac{1}{k},\;\;B>\frac{1}{k} \end{array}$$

are satisfied. Then, every positive solution (*x*_*n*_, *y*_*n*_) of (1) is satisfied, for *n* = *k* + 1, *k* + 2,…

(8) $$\begin{array}{c} A\leq x_{n}\leq \frac{1}{{\left(kB\right)}^{n-k}}\left(x_{k}-\frac{kAB}{kB-1}\right)+\frac{kAB}{kB-1}\\ B\leq y_{n}\leq \frac{1}{{\left(kA\right)}^{n-k}}\left(y_{k}-\frac{kAB}{kA-1}\right)+\frac{kAB}{kA-1}. \end{array}$$

**Proof**

Let {(*x*_*n*_, *y*_*n*_)} be a positive solution of (1). Since *x*_*n*_ > 0 and *y*_*n*_ > 0 for all *n* ≥ 1, (1) implies that

(9) $$\begin{array}{c} x_{n}\geq A,\;y_{n}\geq B,\;n=1,2,3,\ldots \end{array}$$

Moreover, using (1) and (9), we have

(10) xn≤A+1kBxn−1,  yn≤B+1kAyn−1,n=k+1,k+2,….

Let *v*_*n*_, *w*_*n*_ be the solution of the system, respectively,

(11) $$\begin{array}{c} v_{n}=A+\frac{1}{kB}v_{n-1},\;w_{n}=B+\frac{1}{kA}w_{n-1},\;n\geq k+1, \end{array}$$

such that

(12) $$\begin{array}{c} v_{i}=x_{i},\;w_{i}=y_{i},\;i=1,2,\ldots ,k. \end{array}$$

We prove by induction that

(13) $$\begin{array}{c} x_{n}\leq v_{n},\;y_{n}\leq w_{n},\;n\geq k+1. \end{array}$$

Suppose that (13) is true for *n* = *m* ≥ *k* + 1. Then, from (10), we get

(14) $$\begin{array}{c} x_{m+1}\leq A+\frac{1}{kB}x_{m}\leq A+\frac{1}{kB}v_{m}=v_{m+1},\;\\ y_{m+1}\leq B+\frac{1}{kA}y_{m}\leq B+\frac{1}{kB}w_{m}=w_{m+1}. \end{array}$$

Therefore, (13) is true. From (11) and (12), we have

(15) vn=1kBn−kxk−kABkB−1+kABkB−1,wn=1kAn−kyk−kABkA−1+kABkA−1,n≥k.

Then, from (9), (13), and (15), the proof of the relation (8) follows immediately.

**Theorem 2 .**

Consider the system of difference equation (1). If relation (7) is satisfied, then the following statements are true.

(i) Equation (1) has a unique positive equilibrium (*x*, *y*) given by
  (16) $$\begin{array}{c} x=\frac{k^{2}AB-1}{k\left(kB-1\right)},\;\;y=\frac{k^{2}AB-1}{k\left(kA-1\right)}. \end{array}$$
(ii) Every positive solution (*x*_*n*_, *y*_*n*_) of system (1) tends to the positive equilibrium (*x*, *y*) of (1) as *n* → *∞*.

**Proof**

(i) Let *x* and *y* be positive numbers such that

(17) $$\begin{array}{c} x=A+\frac{x}{ky},\;\;y=B+\frac{y}{kx}. \end{array}$$

Then, from (7) and (17), we have that the positive solution (*x*, *y*) is given by (16). This completes the proof of Part (i).

(ii) From (1) and (8), we have

(18) $$\begin{array}{c} \underset{n\to \infty }{\lim}\sup x_{n}=L_{1},\;\;\underset{n\to \infty }{\lim}\inf x_{n}=l_{1},\\ \underset{n\to \infty }{\lim}\sup y_{n}=L_{2},\;\;\underset{n\to \infty }{\lim}\inf y_{n}=l_{2}, \end{array}$$

where *l*_*i*_, *L*_*i*_ ∈ (0, *∞*),  *i* = 1,2. Then, from (1) and (18), we get

(19) $$\begin{array}{c} L_{1}\leq A+\frac{L_{1}}{kl_{2}},\;\;l_{1}\geq A+\frac{l_{1}}{kL_{2}},\;\\ L_{2}\leq B+\frac{L_{2}}{kl_{1}},\;\;l_{2}\geq B+\frac{l_{2}}{kL_{1}}, \end{array}$$

from which we have

(20) $$\begin{array}{c} L_{1}\left(kB-1\right)\leq l_{2}\left(kA-1\right),\;\;L_{2}\left(kA-1\right)\leq l_{1}\left(kB-1\right). \end{array}$$

Then, relations (7) and (20) imply that *L*_1_ *L*_2_ ≤ *l*_1_ *l*_2_, from which it follows that

(21) $$\begin{array}{c} L_{1}L_{2}=l_{1}l_{2}. \end{array}$$

We claim that

(22) $$\begin{array}{c} L_{1}=l_{1},\;\;L_{2}=l_{2}. \end{array}$$

Suppose on the contrary that *l*_1_ < *L*_1_. Then, from (21), we have *L*_1_ *L*_2_ = *l*_1_ *l*_2_ < *L*_1_ *l*_2_ and so *L*_2_ < *l*_2_ which is a contradiction. So *L*_1_ = *l*_1_. Similarly, we can prove that *L*_2_ = *l*_2_. Therefore, (22) is true. Hence, from (1) and (22), there exist the lim⁡*x*_*n*_ and lim⁡*y*_*n*_, as *n* → *∞* and

(23) $$\begin{array}{c} \underset{n\to \infty }{\lim}x_{n}=x,\;\;\underset{n\to \infty }{\lim}y_{n}=y, \end{array}$$

where (*x*, *y*) is the unique positive equilibrium of (1). This completes the proof of Part (ii). The proof of Theorem 2 is completed.

**Theorem 3 .**

Consider the system of difference equation (1). If relation (7) is satisfied and assuming that

(24) $$\begin{array}{c} \frac{k^{2}AB-1}{kA-1}+\frac{k^{2}AB-1}{kB-1}<1, \end{array}$$

then the unique positive equilibrium (*x*, *y*) is locally asymptotically stable.

**Proof**

From Theorem 2, the system of difference equation (1) has a unique equilibrium (*x*, *y*). The linearized equation of system (1) about the equilibrium point (*x*, *y*) is

(25) $$\begin{array}{c} \Psi_{n+1}=B\Psi_{n}, \end{array}$$

where Ψ_*n*_ = (*x*_*n*_,…, *x*_*n*−*k*_, *y*_*n*_,…, *y*_*n*−*k*_)^*T*^, and
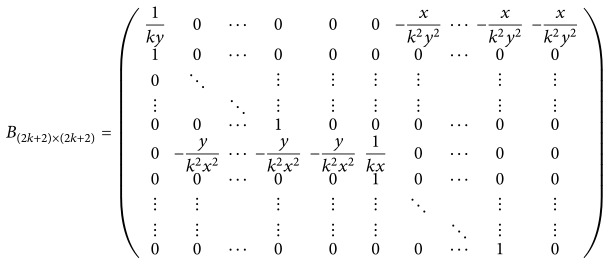
(26) Let *λ*_1_, *λ*_2_,…, *λ*_2*k*+2_ denote the eigenvalues of matrix *B* and let *D* = diag⁡(*d*_1_, *d*_2_,…, *d*_2*k*+2_) be a diagonal matrix, where *d*_1_ = *d*_*k*+2_ = 1,  *d*_*i*_ = *d*_*k*+1+*i*_ = 1 − *iɛ*  (*i* = 2,…, *k* + 1), and

(27) $$\begin{array}{c} 0<ɛ<\min\left\{\frac{1}{k+1}\left(1-\frac{x+y}{ky^{2}}\right),\;\;\frac{1}{k+1}\left(1-\frac{x+y}{kx^{2}}\right)\right\}. \end{array}$$

Clearly, *D* is invertible. Computing matrix *DBD* ^−1^, we obtain that
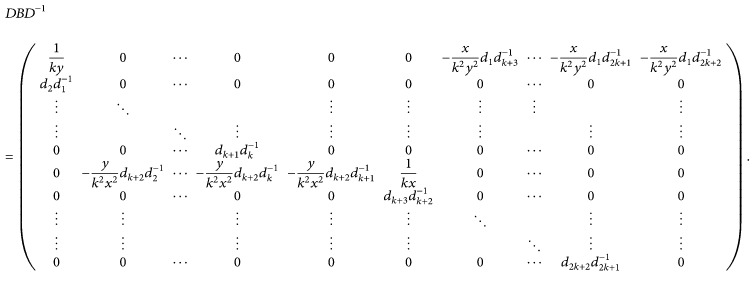
(28)From *d*_1_ > *d*_2_ > ⋯>*d*_*k*+1_ > 0 and *d*_*k*+2_ > *d*_*k*+3_ > ⋯>*d*_2*k*+2_ > 0, imply that

(29) $$\begin{array}{c} d_{2}d_{1}^{-1}<1,\;\;d_{3}d_{2}^{-1}<1,\ldots ,d_{k+1}d_{k}^{-1}<1,\;\\ d_{k+3}d_{k+2}^{-1}<1,\ldots ,d_{2k+2}d_{2k+1}^{-1}<1. \end{array}$$

Furthermore, noting (7), (24), and (27), we have

(30) 1ky+xk2y2d1dk+3−1+⋯+xk2y2d1d2k+2−1  =1ky+xk2y211−2ɛ+⋯+11−(k+1)ɛ  <1ky+xky211−k+1ɛ<1,1kx+yk2x2dk+2d2−1+⋯+yk2x2dk+2dk+1−1  =1kx+yk2x211−2ɛ+⋯+11−(k+1)ɛ  <1kx+ykx211−k+1ɛ<1.

It is well known that *B* has the same eigenvalues as *DBD* ^−1^; we have that

(31) max⁡1≤i≤2k+2λi≤DBD−1∞=max⁡+yk2x211−2ɛ+⋯+11−(k+1)ɛd2d1−1,…,dk+1dk−1,dk+3dk+2−1,…,d2k+2d2k+1−1,1ky    +xk2y211−2ɛ+⋯+11−k+1ɛ,1kx    +  yk2x211−2ɛ+⋯+11−(k+1)ɛ<1.

This implies that the equilibrium (*x*, *y*) of (1) is locally asymptotically stable.

Combining Theorem 2 with Theorem 3, we obtain the following theorem.

**Theorem 4 .**

Consider the system of difference equation (1). If relations (7) and (24) are satisfied, then the unique positive equilibrium (*x*, *y*) is globally asymptotically stable.

## 3. Some Numerical Examples

In order to illustrate the results of the previous sections and to support our theoretical discussions, we consider several interesting numerical examples in this section. These examples represent different types of qualitative behavior of solutions to nonlinear difference equations and system of nonlinear difference equations.

**Example 1 .**

**Figure 1:**
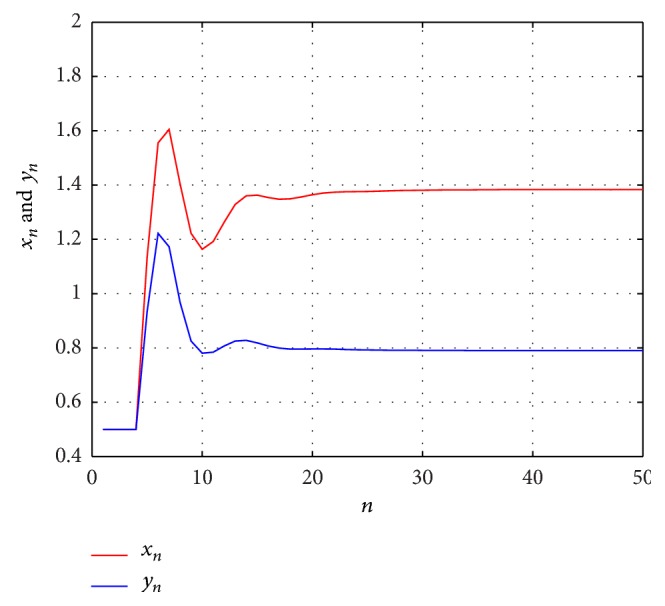
The dynamics of system (32).

Consider the following difference equations:

(32) $$\begin{array}{c} x_{n+1}=0.8+\frac{x_{n}}{y_{n-1}+y_{n-2}+y_{n-3}},\;\\ y_{n+1}=0.6+\frac{y_{n}}{x_{n-1}+x_{n-2}+x_{n-3}}, \end{array}$$

with the initial values *x*_−*i*_ = *y*_−*i*_ = 0.5  (*i* = 1,2, 3). Then, the solution (*x*_*n*_, *y*_*n*_) of system (32) is bounded and persists and the system has a unique equilibrium (*x*, *y*) = (1.3833,0.7905) which is globally asymptotically stable (see Figure 1).

**Example 2 .**

**Figure 2:**
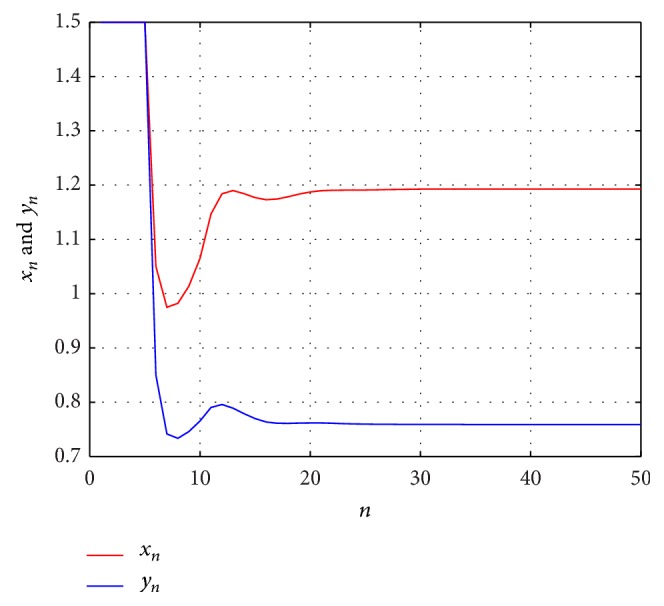
The dynamics of system (33).

Consider the following difference equations:

(33) $$\begin{array}{c} x_{n+1}=0.8+\frac{x_{n}}{y_{n-1}+y_{n-2}+y_{n-3}+y_{n-4}},\;\\ y_{n+1}=0.6+\frac{y_{n}}{x_{n-1}+x_{n-2}+x_{n-3}+x_{n-4}}, \end{array}$$

with the initial values *x*_−*i*_ = *y*_−*i*_ = 1.5  (*i* = 1,2, 3,4). Then, the solution (*x*_*n*_, *y*_*n*_) of system (33) is bounded and persists and the system has a unique equilibrium (*x*, *y*) = (1.1929,0.7591) which is globally asymptotically stable (see Figure 2).

## 4. Conclusion

In this paper, we study the dynamics of a system of high order difference equation

(34) xn+1=A+xn∑i=1kyn−i,  yn+1=B+yn∑i=1kxn−i    n=0,1,…,  k∈1,2,….

It concluded that, under condition *A* > 1/*k*, *B* > 1/*k*, the positive solution (*x*_*n*_, *y*_*n*_) of this system is bounded and persists; moreover, if ((*k* ^2^ *AB* − 1)/(*kA* − 1)) + ((*k* ^2^ *AB* − 1)/(*kB* − 1)) < 1, it converges asymptotically the unique equilibrium (*x*, *y*).

We conclude the paper by presenting the following open problem.

*Open Problem*. Consider the system of difference equation (1) with *A* ≤ 1/*k* and *B* ≤ 1/*k*. Find the set of all initial conditions that generate bounded solutions. In addition, investigate global behavior of these solutions.

## References

[1] DeVault R., Ladas G., Schultz S. W. (1998). On the recursive sequence *x*
_*n*+1_ = *A*/*x*
_*n*_ + 1/*x*
_*n*−2_. *Proceedings of the American Mathematical Society*.

[2] Abu-Saris R. M., DeVault R. (2003). Global stability of *y*
_*n*+1_ = *A* + *y*
_*n*_/*y*
_*n*−*k*_. *Applied Mathematics Letters*.

[3] Papaschinopoulos G., Schinas C. J. (1998). On a system of two nonlinear difference equations. *Journal of Mathematical Analysis and Applications*.

[4] Zhang Q., Yang L., Liao D. (2012). Global asymptotic behavior of positive solutions to the system of rational difference equations. *Journal of Southwest University*.

[5] Zhang Q., Yang L., Liu J. (2012). Dynamics of a system of rational third-order difference equation. *Advances in Difference Equations*.

[6] Amleh A. M., Grove E. A., Ladas G., Georgiou D. (1999). On the recursive sequence *x*
_*n*+1_ = *A* + (*x*
_*n*−1_/*x*
_*n*_). *Journal of Mathematical Analysis and Applications*.

[7] He W., Li W., Yan X. (2004). Global attractivity of the difference equation *x*
_*n*+1_ = *a* + *x*
_*n*−*k*_/*x*
_*n*_. *Applied Mathematics and Computation*.

[8] DeVault R., Ladas G., Schultz S. W. (1998). Necessary and sufficient conditions the boundedness of *x*
_*n*+1_ = *A*/*x*
_*n*_
^*p*^ + *B*/*x*
_*n*−1_
^*q*^. *Journal of Difference Equations and Applications*.

[9] Elaydi S. N. (1996). *An Introduction to Difference Equations*.

[10] Agarwal R. P., Li W., Pang P. Y. H. (2002). Asymptotic behavior of a class of nonlinear delay difference equations. *Journal of Difference Equations and Applications*.

[11] Kocic V. L., Ladas G. (1993). *Global Behavior of Nonlinear Difference Equations of Higher Order with Applications*.

[12] Kulenonvic M. R. S., Ladas G. (2002). *Dynamics of Second Order Rational Difference Equations with Open Problems and Conjectures*.

[13] Li W., Sun H. (2005). Dynamics of a rational difference equation. *Applied Mathematics and Computation*.

[14] Su Y., Li W. (2005). Global attractivity of a higher order nonlinear difference equation. *Journal of Difference Equations and Applications*.

[15] Hu L., Li W. (2007). Global stability of a rational difference equation. *Applied Mathematics and Computation*.

[16] Ibrahim T. F. (2012). Two-dimensional fractional system of nonlinear difference equations in the modelling competitive populations. *International Journal of Basic & Applied Sciences*.

[17] Ibrahim T. F. (2012). Closed form solution of a symmetric competitive system of rational difference equations. *Studies in Mathematical Sciences*.

[18] Ibrahim T. F., Zhang Q. (2013). Stability of an anti-competitive system of rational difference equations. *Archives des Sciences*.

